# Metagenomic insights into the dominant Fe(II) oxidizing *Zetaproteobacteria* from an iron mat at Lō´ihi, Hawai´l

**DOI:** 10.3389/fmicb.2013.00052

**Published:** 2013-03-19

**Authors:** Esther Singer, John F. Heidelberg, Ashita Dhillon, Katrina J. Edwards

**Affiliations:** ^1^Department of Earth Sciences, University of Southern CaliforniaLos Angeles, CA, USA; ^2^Department of Biological Sciences, University of Southern CaliforniaLos Angeles, CA, USA; ^3^Genzyme CorporationAllston, MA, USA

**Keywords:** iron oxidation, Lō´ihi Seamount, molybdopterin oxidoreductase, Rubisco, *Zetaproteobacteria*, hydrothermal vents, sulfide oxidation

## Abstract

*Zetaproteobacteria* are among the most prevalent Fe(II)-oxidizing bacteria (FeOB) at deep-sea hydrothermal vents; however, knowledge about their environmental significance is limited. We provide metagenomic insights into an iron mat at the Lō´ihi Seamount, Hawai´l, revealing novel genomic information of locally dominant *Zetaproteobacteria* lineages. These lineages were previously estimated to account for ~13% of all local *Zetaproteobacteria* based on 16S clone library data. Biogeochemically relevant genes include nitrite reductases, which were previously not identified in *Zetaproteobacteria*, sulfide:quinone oxidases, and ribulose-1,5-bisphosphate carboxylase (RuBisCo). Genes assumed to be involved in Fe(II) oxidation correlate in synteny and share 87% amino acid similarity with those previously identified in the related *Zetaproteobacterium* *Mariprofundus ferrooxydans* PV-1. Overall, *Zetaproteobacteria* genes appear to originate primarily from within the *Proteobacteria* and the Fe(II)-oxidizing *Leptospirillum* spp. and are predicted to facilitate adaptation to a deep-sea hydrothermal vent environment in addition to microaerophilic Fe(II) and H_2_S oxidation. This dataset represents the first metagenomic study of FeOB from an iron oxide mat at a deep-sea hydrothermal habitat.

## INTRODUCTION

Microbial Fe(II) oxidation is widespread in the deep-sea and is thought to play a significant role in rock and mineral weathering (Edwards et al., 2004). The microbial influence on the global iron cycle, and consequently on other linked biogeochemical cycles, such as carbon, has remained poorly understood, although several studies have recently started to investigate key habitats with large potential to host Fe(II)-oxidizing bacteria (FeOB; Edwards et al., 2003, 2004; Rassa et al., 2009; Emerson and Moyer, 2010; Emerson, 2012). Among these habitats are 125,000 seamounts worldwide, which often host extensive hydrothermal vent systems and Fe-rich mats of microbial origin (Wessel et al., 2010; McAllister et al., 2011).

Compared to the most commonly studied marine hydrothermal systems, located at mid-ocean ridge (MOR) spreading centers, the Lō´ihi Seamount hydrothermal system has unique fluid chemistries. For example, vent fluids at Lō´ihi are highly enriched in CO_2_, CH_4_, NH_4_, PO_4_, Fe, and Mn, but depleted in H_2_S (Karl et al., 1988; Sedwick et al., 1992; Wheat et al., 2000; Edwards et al., 2004; Glazer and Rouxel, 2009). The high concentration of dissolved CO_2_ buffers the hydrothermal fluids at a lower pH (5.3–5.5) compared to what is most commonly observed at MOR systems (Sedwick et al., 1992; Edwards et al., 2004). The low sulfide concentration results in high hydrothermal Fe concentrations and extensive iron oxide mats by comparison to many MOR systems, where Fe predominately occurs as FeS instead. The summit of Lō´ihi intersects the Oxygen Minimum Zone (OMZ). The low O_2_ concentration in bottom seawater at ~1,000 m depth (O_2_ ~50μM) associated with this OMZ and the high Fe concentrations (up to 500 μM) in warm hydrothermal fluids (below 100°C) present beneficial conditions for FeOB, who have to compete with the abiotic oxidation of Fe (Glazer and Rouxel, 2009).

The Lō´ihi Seamount supports abundant FeOB, and is dominated by Fe(II)-oxidizing *Zetaproteobacteria*, as shown in various studies (Emerson and Moyer, 2002; Rassa et al., 2009). The isolation of the first *Zetaproteobacterium* *Mariprofundus ferrooxydans* PV-1 from an iron mat at a cool (23°C) diffuse vent site at the Lō’ihi Seamount, has initiated evaluation of the ecological significance of this class in biocorrosion (Emerson et al., 2007; Weiss et al., 2007; Singer et al., 2011). Since then *Zetaproteobacteria* have been predominantly found in diverse marine environments and their involvement in microbially mediated Fe(II) oxidation is widely accepted (Handley et al., 2010; Dang et al., 2011; Meyer-Dombard et al., 2012). Continued discoveries of *Zetaproteobacteria* in marine Fe(II) oxidizing niches raise the question whether *Zetaproteobacteria* could be the dominant marine FeOB. However, cultivation of these FeOB present various difficulties to date. Molecular and functional assays to study the biogeochemistry and ecology of Fe(II) oxidation, the molecular mechanism of Fe(II) oxidation, and the cultivation of environmentally representative groups of *Zetaproteobacteria* all have remained elusive and have consequently called for cultivation-independent molecular techniques. Full-length *Zetaproteobacteria* 16S rRNA sequences have so far been published from 11 regions in the world oceans and show that this class appears to follow a strong biogeographic distribution (McAllister et al., 2011). The distribution of operational taxonomic units (OTUs) appears to be more strongly correlated with geographic occurrence than with environmental parameters, such as temperature, pH, or total Fe concentration (McAllister et al., 2011). The genome of *Mariprofundus ferrooxydans* PV-1 has provided insights into the genomic underlyings of Fe(II)-oxidizing *Zetaproteobacteria *(Singer et al., 2011), but analysis of FeOB-associated iron oxyhydroxide stalks suggests that PV-1 plays a minor role at most sites (Emerson and Moyer, 2010). This study discusses the (meta-)genomic content of new *Zetaproteobacteria* lineages dominant at the Lō´ihi Seamount, Loh clone SPL-4, and Loh clone SPL-7, delivers genomic and proteomic comparisons to PV-1, and attempts to evaluate the environmental potential of *Zetaproteobacteria* at Fe-rich seamounts in the global oceans.

## MATERIALS AND METHODS

### ENVIRONMENTAL SAMPLE SOURCE

Iron oxide-coated microbial biomats were collected by suction sampler using the submersible vehicle *Pisces V* at the Spillway site (Marker 34; 18.91 N, 155.26 W, 1271.95 m, *T*_max_ = 63°C) at the Lō´ihi Seamount, Hawai´I, in 2003. DNA was extracted using a phenol-chloroform extraction and purified using cesium chloride density gradient centrifugation as described by (Moore and Dowhan, 2002). The extracted and purified DNA was used to construct a library of ~8,000 fosmids (each ~35 kbp) with the Copy Control^TM^ Fosmid Library Production kit (Epicentre, Madison, WI, USA) according to the manufacturer’s protocol and stored at -80°C. DNA was extracted at random from 384 fosmids (total library size ≈13.4 Mbp) with the BACMAX^TM^ DNA Purification Kit (Epicentre, Madison, WI, USA) in 2011. The extraction protocol was amended by extending the incubation with the Plasmid DNA Safe mix from 20 min to 24 h under the addition of extra Plasmid-Safe DNase and ATP to minimize contaminating genomic DNA from the *Escherichia coli* fosmid vector.

### SEQUENCING AND ANNOTATION

DNA concentrations from individual fosmids were measured on a Qubit 2.0 Fluorometer and pooled at equal molar amounts. The pooled DNA was sequenced using conventional whole-genome shotgun sequencing on a Roche 454 GS FLX Titanium sequencer at the Core Genomics Center at the University of Pennsylvania and yielded 1,492,332 reads (~816 Mbp). Quality control was performed with Prinseq v0.20.1 (maximum length: 450 bp; minimum quality: 25; maximum homopolymer length: 9 bp; maximum N-tail: 1 bp) (Schmieder and Edwards, 2011) and fosmid vectors were discarded with a custom biopython script (Cock et al., 2009). The remaining metagenome (~423 Mbp) was assembled with Mira using settings: *de novo*, genome, accurate, 454 (Chevreux et al., 1999) and resulted in 3,988 contigs. These 3,988 contigs were further assembled using Geneious Pro v. 5.4.6 (Drummond et al., 2011) leading to 2,865 final contigs with minimum length of 200 bp, average length of 1,956 bp, and average coverage of >25×. Annotation of these final 2,865 contigs was performed on the RAST (Rapid Annotation using Subsystem Technology) server version 4.0 (Aziz et al., 2008). Manual curation was performed with the SEED Viewer v. 2.0 (Overbeek, 2005). rRNA sequences were predicted using BLASTN in CAMERA (Community cyberinfrastructure for Advanced Microbial Ecology Research and Analysis; Sun et al., 2011). Alignment and phylogenetic tree construction of 16S rRNA sequences were performed with the Geneious aligner and PHYML tree builder using the Jukes-Cantor substitution model and 100 bootstraps in Geneious (Guindon and Gascuel, 2003). This Whole-Genome Shotgun project has been deposited at DDBJ/EMBL/GenBank under the accession AMFO00000000. The version described in this paper is the first version, AMFO0-1000000.

### PHYLOGENETIC BINNING

RAST-annotated genes were aligned against the NCBI non-redundant database using the BLASTX algorithm (E 10^-^^5^) for community structure analysis. Final contigs of *Zetaproteobacteria* origin were determined by alignment against the genome of *Mariprofundus ferrooxydans* PV-1 (AATS01000000) using the BLASTX algorithm: Genes with best BLASTX matches to the genome of PV-1 average to 62.15% average amino acid identity (AAid) (E 10^-^^5^). On this basis we selected all genes with BLASTX hits of ≥60% AAid to PV-1 proteins to be preliminarily binned as *Zetaproteobacteria* genes. Contigs exclusively harboring best BLASTX matches to PV-1 homologs at ≥60% AAid were classified as *Zetaproteobacteria* contigs without further analysis. Contigs including genes homologous to PV-1 genes, but also to other bacterial classes were only included in our *Zeta*-subset if they passed the following requirements: (1) *Zetaproteobacteria* genes dominated in overall number and AAid or (2) remaining non-*Zetaproteobacteria *genes could not be attributed to a single phylogenetic class, which therefore excluded the possibility of chimeras (Treangen et al., 2011). This screening analysis resulted in 853 genes on 86 contigs with 85.57% ANI to PV-1. Ambiguous contigs, which may still belong to the genome of this *Zetaproteobacterium*, but did not pass our screening filters discussed above, were not included in the discussion of this study.

### DNA AND PROTEIN SEQUENCE ANALYSIS

DNA sequence synteny was evaluated with the Artemis Comparison Tool (ACT; Carver et al., 2005). Protein subcellular localization analysis was performed with PSORT (Nakai and Horton, 1999) and Gneg-mPLoc (Shen and Chou, 2010). Signal peptides and distinction between the general export pathway (Sec) and the twin-arginine translocase (Tat) mechanism were predicted on the basis of Hidden Markov Models (HMMs) using PRED–TAT (Bagos et al., 2010). Motif analysis was performed in the Pfam database using MOTIF Search on the GenomeNet network of Kyoto University available at (http://www.genome.jp).

## RESULTS

### PHYLOGENY

**FIGURE 1:**
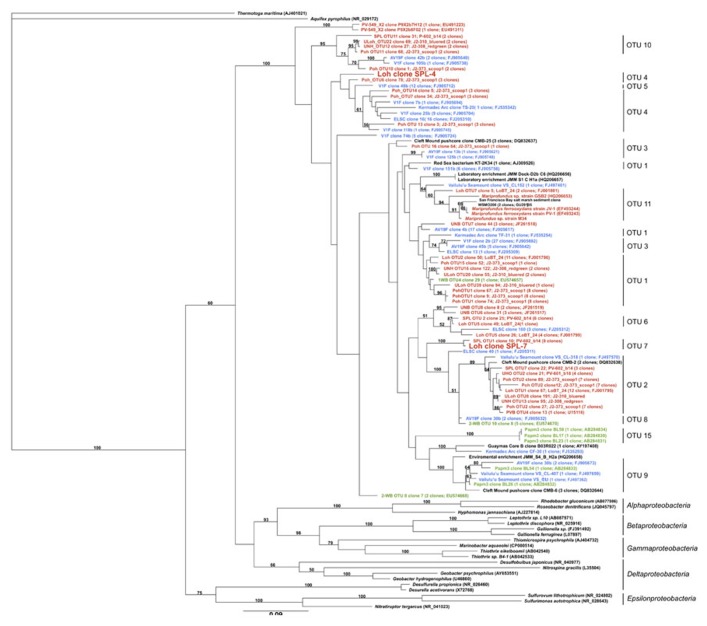
**Maximum-likelihood tree with 100 bootstrap cycles of full-length 16S rRNA gene sequences of all known *Zetaproteobacteria* species to date and representatives from other *Proteobacteria* classes as stated in (McAllister et al., 2011)**. Coloring is by geographic origin within the Pacific Ocean: red – Lō´ihi Seamount, blue – Vailulu’u Seamount/Tonga Arc/East Lau Spreading Center/Kermadec Arc, green – Southern Mariana Trough. GenBank accession numbers for published sequences are shown in parentheses. The scale bar represents 9 nucleotide substitutions per 100 positions.

In total, the dataset harbors five environmental 16S rRNA sequences (>500 bp), two of which are nearly full-length *Zetaproteobacteria* sequences (**Table 1**; **Figure 1**). Based on this phylogenetic marker, *Zetaproteobacteria* are well represented at Marker 34, Lō´ihi, which is in line with earlier studies based on polymerase chain reaction (PCR)-dependent 16S rRNA analyses (Moyer et al., 1995; Emerson and Moyer, 2002; Rassa et al., 2009). rRNA sequences from other FeOB were not detected. On the basis of their isolation from the Spillway (SPL) site at Lō´ihi, their phylogenetic placement in OTU groups 4 and 7, and because they have not been cultured to date, we named the new *Zetaproteobacteria* lineages Loh clone SPL-4 and Loh clone SPL-7 (**Figure 1**). All *Zetaproteobacteria* genes presented here are assumed to belong to these two lineages.

**Table 1:** Best BLASTN hits of 16S rRNA sequences (>500 bp) in the Lō´ihi iron mat metagenome (retrieved 01/2013).

Closest relative in GenBank	Class	Accession no.	Contig	Length [bp]
SPL OTU 1 clone 10	*Zetaproteobacteria*	JF320745	592	1,415
Uncultured bacterium clone Poh_5	*Zetaproteobacteria*	JF320730	1645	1,427
*Methylomicrobium alcaliphilum*	*Gammaproteobacteria*	FO082060	202	1,147
*Methylobacter psychrophilus*	*Gammaproteobacteria*	NR_025016	2858	770
Uncultured bacterium clone Ld1-14	*Actinobacteria*	GQ246409	2406	1,485

**Zetaproteobacteria* relatives are marked in bold letters. 16S rRNA sequences from potential fosmid vector contamination (*Escherichia coli*) are not listed here.*

16S rRNA genes from SPL-4 and SPL-7 are 91% identical to each other, and 90 and 93% identical to the 16S rRNA gene of *Mariprofundus ferrooxydans* PV-1, respectively. Closest relatives from OTUs 4 and 7 have been sampled in the Northern and Southern Pacific, but were previously not considered dominant OTUs at Lō´ihi (McAllister et al., 2011). Clones of OTU 7 have so far only been detected at the Lō´ihi Seamount (Marker 34) representing ~5% of all local *Zetaproteobacteria*; clones from OTU 4 have been isolated from Markers 48 and 57 at Lō´ihi (~8% of local *Zetaproteobacteria*), the Tonga Arc, and the East Lau Spreading Center, and so far show a wider distribution in the Pacific oceans compared to clones from OTU 7 according to (McAllister et al., 2011). Although the biodiversity and biogeography study by McAllister et al. (2011) is the most comprehensive to date, it is important to note that their findings completely rely on results from clone libraries, the results of which are likely to be significantly biased, for instance by *Taq* DNA polymerase errors and PCR template concentrations (Chandler et al., 1997; Acinas et al., 2005). Our findings could hence portrait genomic traits that are shared by other Lō´ihi strains and may be applicable to more ubiquitous *Zetaproteobacteria*.

### BIOGEOCHEMICALLY RELEVANT GENES

*Zetaproteobacteria* genes were analyzed with respect to metabolic potential and environmental significance to the iron mat environment at Lō´ihi. We also attempted a broader description of the nature of the *Zetaproteobacteria* by comparative (meta-)genomics with the genome of *Mariprofundus ferrooxy-dans* PV-1.

### NEW *Zetaproteobacteria* GENE FUNCTIONS

**FIGURE 2:**
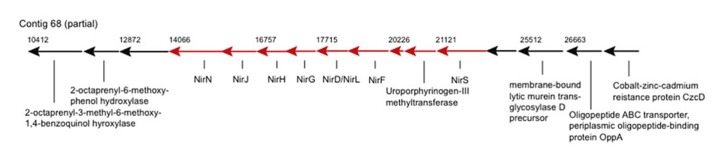
**Genes encoding for nitrite reductase and heme d_**1**_ biosynthesis are represented by arrows in red**. Numbers denote starting positions (bp) of genes within the contig.

Genes of potential biogeochemical relevance, which have not been described for a *Zetaproteobacterium* before, encode for nitrite reduction (contigs 68 and 2306, **Figure 2**). The nitrite reduction gene cluster on contig 68 is similar in gene content and synteny to those in the genomes of denitrifying *Thiobacillus denitrificans*, *Pseudomonas aeruginosa*, *P. stutzeri*, and *P. denitrificans *(Rinaldo and Cutruzzolà, 2007), e.g., NirS-encoded cytochrome *cd*_1_ nitrite reductase (cd_1_NIR) shares 82% amino acid similarity (AASim) with nitrite reductase from the versatile *Thiobacillus denitrificans* ATCC 25259 (gb∣AAZ96030.1). Contig 2306 encodes copper-containing nitrite reductase NirK (CuNIR) with 80% and 70% AASim to NirK in the ammonia-oxidizing *Nitrosococcus halophilus* and *Nitrosomonas europaea*, respectively. *nirK* occurs in a cluster next to genes encoding for cytochromes *c*, which have 68% and 66% AASim to NcgB and NcgC from *Nitrosomonas europaea*, as well as next to two genes encoding for multicopper oxidases type 3, which have 63% and 68% AASim to NcgA.

The two types of dissimilatory NiRs containing either heme *cd*_1_ or two types of Cu centers as prosthetic groups, encoded by *nirS* and *nirK*, respectively, have not been shown to coexist in the same bacterial species, assuming that both encode nitrite reducing activity (Cutruzzolà, 1999; Jones et al., 2008). Hence either nitrite reductases are not conserved among different *Zetaproteobacteria* spp. or *nirK* and *ncgABC* are rather involved in nitrite detoxification, such as in *Nitrosomonas europaea* (Beaumont et al., 2002). Nevertheless, nitrite reduction may be coupled to Fe(II) oxidation and could render certain *Zetaproteobacteria* spp. facultative anaerobes. This would allow these strains to inhabit a wider range of environments than PV-1, which is known to be a strict microaerophile and can typically acquire energy only at <5% of air-saturated values (Emerson and Merrill Floyd, 2005; Weiss et al., 2007).

It is difficult to infer evolutionary paths of nitrite reductases, because neither *nirS* nor *nirK* are reliable phylogenetic markers (with *nirS* following 16S rRNA phylogenies more congruently). Since both, *nirS* and *nirK* in the *Zetaproteobacteria* genes share most comparable AASim to respective functional genes within the *Gammaproteobacteria*, it remains elusive, which of these types of nitrite reductase is more representative for the *Zetaproteobacteria* and whether one or both types of nitrite reductases were introduced by horizontal gene transfer (HGT).

Nitrate and nitrite reduction are encoded by various other organisms in this Lō´ihi mat environment. Among these organisms are the hydrothermal vent-adapted, thermophilic, strictly aerobic *Marinithermus hydrothermalis*, the sulfur-oxidizing endosymbionts of *Riftia pachyptila* (vent Ph05) and of *Tevnia jerichonana* (vent Tica), which both have very similar physiologies (Gardebrecht et al., 2011), the purple sulfur bacterium *Thiocystis violascens* DSM 198 (Kämpf and Pfennig, 1980), and the widely distributed *Thiobacillus denitrificans*, a facultative anaerobe, which couples inorganic sulfur oxidation as well as anaerobic oxidation of Fe(II) to denitrification at circumneutral pH (Beller et al., 2006). It appears that most nitrate/nitrite reducing organisms in our dataset are adapted to hydrothermal vent life either following a free-living or endosymbiotic lifestyle and many possess the ability to oxidize a form of sulfur, such as the *Zetaproteobacteria*, as well. Since the iron mat environment provides aerobic and anaerobic niches, the ability to reduce nitrate/nitrite is consequently a beneficial trait that can easily be coupled to the oxidation of inorganic compounds, such as sulfide and iron, and therefore allow survival under dynamic environmental conditions.

Besides nitrite reduction, new *Zetaproteobacteria* gene functions include antibiotic biosynthesis monooxygenase (contig 285), several exodeoxyribonucleases I (contigs 2596, 2709, 280, 68), and three transposases. Otherwise, the genes available from PV-1 and the SPL-strains are fairly comparable in function and abundance. The gene novelty in the *Zetaproteobacteria* genes fit with the lifestyle of facultative anaerobic microorganisms, which inhabit mats at an Fe-rich hydrothermal vent environment and are frequently exposed to contact with other bacteria. Different *Zetaproteobacteria* lineages may adapt to their exact niche via acquisition of diverse survival-enhancing genes, e.g., specific antibiotic biosynthesis or efflux, or more/less heavy metal efflux pumps encoding genes, however, the overall main metabolic potentials appear similar between strains.

### COMPARATIVE GENOMICS WITH *Mariprofundus ferrooxydans* PV-1

#### Iron oxidation

**FIGURE 3:**
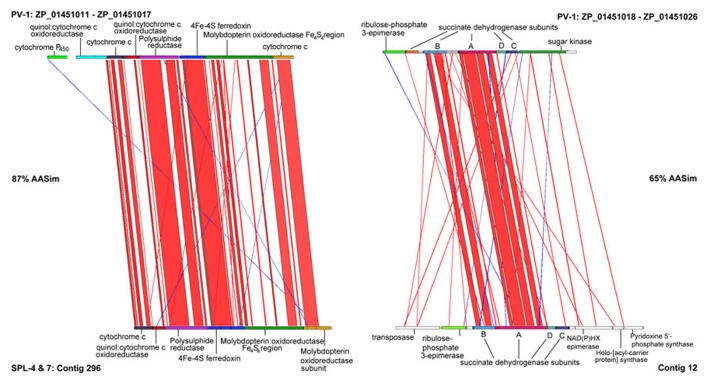
**Artemis Comparison Tool (ACT) analysis of gene neighborhoods around Molybdopterin oxidoreductase Fe_**4**_S_**4**_ region (MobB) on contigs 296 and 12 from the SPL *Zetaproteobacteria* and in *Mariprofundus ferrooxydans* PV-1**.

The first *Zetaproteobacteria* gene candidates assumed to be involved in neutrophilic microaeorophilic Fe(II) oxidation were detected via protein extraction from an Fe(II)-oxidizing PV-1 cell culture (Singer et al., 2011). The extracted molybdopterin oxidoreductase Fe_4_S_4_ region (MobB) and most of the surrounding gene cluster (ZP_01451010- ZP_01451022) was also identified in our dataset (contigs 12, 296) and shows that gene synteny is well conserved, however, split over two contigs (**Figure 3**). Cytochromes and MobB (contig 296) are more conserved than succinate dehydrogenases (contig 12).

**FIGURE 4:**
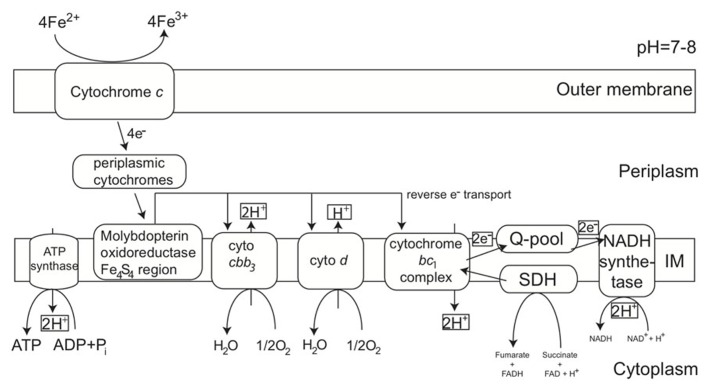
**Conceptual iron oxidation model at neutral pH in *Zetaproteobacteria* revised from (Singer et al., 2011)**. Proteins potentially involved in energy acquisition via Fe(ll) oxidation through the outer and inner membrane as predicted from genomic and protein structure and localization analysis. Besides Molybdopterin oxidoreductase Fe_4_S_4_ region, proteins extracted from Fe(ll) oxidizing *Zetaproteobacteria* cultures are discussed in (Barco et al., in preparation).

MobB in the SPL-strains shares 82% AASim with MobB in PV-1, while it is 59% and 57% similar to MobB in *Gallionella capsiferriformans* and *Sideroxydans lithotrophicus*, respectively. This indicates that there are sequence and potentially structural differences among MobB within the *Zetaproteobacteria* and in comparison to other FeOB. Both MobB proteins, in PV-1 as well as in the SPL-strains, contain Tat signal sequences and are predicted to harbor transmembrane helices located in the inner membrane. Based on their negative charge at pH 7, the soluble parts of both MobB, including the Fe_4_S_4_ region, are predicted to face the periplasm unlike previously described in (Singer et al., 2011). MobB may therefore accept electrons shuttled from the outer membrane to the periplasm during Fe(II) oxidation as depicted in our revised conceptual iron oxidation model (**Figure 4**). Outer membrane cytochromes are likely involved in the import of electrons from ferrous iron into the cells of FeOB, such as in *Acidithiobacillus ferrooxidans* (Bird et al., 2011). Both, PV-1 and the SPL lineages, also harbor genes encoding type IV biogenesis proteins PilAMNOPQ (contigs 592 and 1144), which may aid in the direct contact of Fe-species, such as in *Geobacter* spp. (Mehta et al., 2005).

As neither transcription factors, nor promoters were found in the immediate gene neighborhood vicinity of the MobB gene cluster, it remains unclear if it is in fact actively transcribed in the organism or if transcription is dependent on other genes, which are elsewhere in the genome, for instance involved in redox sensing. Despite the disconnection between contigs 296 and 12 in the SPL-strains, succinate dehydrogenases may still be part of the electron transport chain shuttling electrons during Fe(II) oxidation, however, may not necessarily be transcribed together with the MobB gene cluster.

#### Carbon fixation

Genes from our SPL lineages encode for Form IAq ribulose-1,5-biphosphate carboxylase (RuBisCo) large subunit (contig 280) with 77% AASim to *Mariprofundus ferrooxydans* PV-1 (ZP_01451219) and two RuBisCo activation proteins CbbO and CbbQ (contig 280), which are 72% and 88% similar to respective genes in PV-1 (ZP_01451217, ZP_01451218), respectively. Form IAq appears predominantly in obligate chemolithotrophs and functions best in niches with medium to low CO_2_ concentrations (0.1–1%) and O_2_ present (Badger and Bek, 2007). Form II RuBisCo proteins, which are present in our dataset, could not be unambiguously allocated to the *Zetaproteobacteria* genes, although RuBisCo large and small chain proteins (contig 135) are 87% and 90% similar to respective proteins encoded on the PV-1 genome (ZP_01453295-96). Associated RuBisCo activation proteins CbbQ and CbbO (contig 135) are 81 and 66% similar to CbbQ (ZP_01453297) and CbbO (ZP_01453298) in PV-1. Form II RuBisCo enzymes have a low discrimination threshold against O_2_ as an alternative substrate, poor affinity for CO_2_, and therefore potentially take over when the organism moves to a high-CO_2_ (1.5%) and low-O_2_ environment (Badger and Bek, 2007).

At Marker 34, temperature differences between ambient seawater (2.6°C) and hydrothermal efflux (27°C) may create turbulent eddies in the water column, which would expose cells to oscillating anaerobic and microaerobic conditions, where CO_2_ levels are variable (ranging from 2 mM to 20 mM) and dependent on positioning within the chemocline interface (Badger and Bek, 2007; Glazer and Rouxel, 2009). Utilization of both forms of RuBisCo proteins could thus enable SPL-4 and SPL-7 to optimize the acquisition of carbon under a wider range of CO_2_ and O_2_ concentrations inside and outside the mat in this dynamic system.

#### Sulfide oxidation

Sulfide oxidation is encoded by a sulfide-quinone reductase (contig 212), which is 83% AASim to sulfide-quinone reductase in PV-1 (ZP_01453072). The presence of a transcriptional regulator two genes further upstream (most closely related to ZP_01451744) suggests that sulfide:quinone oxidoreductase is actively transcribed. Homologs of sulfide-quinone reductases from the SPL-strains are most closely related to genes in *L. ferrooxidans* and other *Leptospirillum* spp., as well as *S. lithotrophicus*. Neither of these organisms was isolated from deep-sea hydrothermal vents, but they are associated with mats dominated by FeOB (Edwards et al., 2000; Emerson et al., 2007; Goltsman et al., 2009).

The Lō´ihi Seamount is mostly deplete of sulfide (e.g., no sulfur phases were observed in 2006), which is why Fe-oxides are the most common form of Fe(III)-minerals (Glazer and Rouxel, 2009). An elevation in dissolved sulfide concentration has been observed at Lō´ihi during an eruption in 1996 (Davis et al., 2003), however, incidents like that represent the exception rather than the norm. It was recently shown that the distribution of *Zetaproteobacteria* OTUs is more dependent on geographic factors, such as distance than environmental chemistry (McAllister et al., 2011). Because sulfide oxidation is an unfavorable metabolism at Lō’ihi the maintenance of sulfide and sulfite oxidation genes should be an evolutionarily unstable strategy. Hence it remains to be determined if all *Zetaproteobacteria* are capable of sulfide oxidation and if these sulfide-quinone reductases are remainders of ancient *Zetaproteobacteria* or have been introduced along with other genes from FeOB, which may experience high sulfide concentrations more frequently.

## DISCUSSION

### THE *Zetaproteobacteria* IN THE GLOBAL OCEANS

This study has provided novel insights into the physiology, ecology, and genetics of novel *Zetaproteobacteria* strains cycling iron and carbon at a deep-sea hydrothermal vent environment. Loh clones SPL-4 and SPL-7 belong to OTUs 4 and 7, which were previously estimated to account for ~13% of the *Zetaproteobacteria* present at the Lō´ihi Seamount based on 16S clone library data (McAllister et al., 2011). In our metagenomic dataset, which was created from samples of the same environment and not amplified prior to sequencing, these strains were dominant. The discrepancy between PCR- and fosmid-based techniques exemplifies the outcome of barely predictable biases, which should be taken into account, especially when studying microbial diversity in Fe(II) oxidizing environments. In addition, the fosmid kits used in our study were accompanied by major difficulties upon insert retrieval and led to a loss of 50% of the library, primarily due to vector contamination (see Methods section). This loss could have been reduced if whole genome sequencing on environmental DNA had been chosen over the use of fosmid libraries. Thereby a significant amount of genomic content missing from our dataset could have possibly been retrieved and would have potentially revealed further molecular fundamentals of the successful *Zetaproteobacteria* lifestyle beyond those discussed in this study. Annotation of *Zetaproteobacteria* genes was difficult sometimes as current databases are skewed toward *Gammaproteobacteria*, however, the combination of screening filters, including BLAST searches, tetranucleotide patterns and taxonomic classification models based on GLIMMER interpolated context models (ICMs) was tested and enables reliable detection of most *Zetaproteobacteria* genes.

Comparative genomics of SPL-4 and SPL7 with *Mariprofundus ferrooxydans* PV-1, show that iron transporters, mat-specific genes, diverse oxygen-level dependent forms of RuBisCo, sulfide:quinone reductases, transmembrane phosphate uptake transporters, and heavy metal efflux pumps are well conserved and support genome-wide relatedness of these deep-sea hydrothermal vent *Zetaproteobacteria* as predicted from their geographical closeness. Furthermore, the SPL-strains also show parallels in metabolic potential and gene relatedness to other hydrothermal vent-native organisms, especially to endosymbionts of *R. pachyptila* and the physiologically similar endosymbiont of *Tevnia jerichonana*. Shared metabolic functions include nitrite reduction, sulfide oxidation, as well as genes typically found in mats, e.g., pili assembly genes. Interesting is the presence of five transposases in the *Zetaproteobacteria* contigs, four of which are most closely related to *Leptospirillum* spp. Unfortunately, these transposases are mostly located next to genes of unknown function and do not reveal information about which (if any) functions are potentially transferred between iron-oxidizing organisms. However, the transposed potential carried between FeOB may be of future research interest as genomic similarities between iron oxidation pathways among FeOB are scarce.

Although PV-1 and the SPL-strains are not closely related on the basis of 16S rRNA, the present genomic data have revealed that key metabolic pathways are often conserved as operon structures. However, gene and protein sequences between the analyzed lineages can be evolutionary divergent, for example MobB, assumed to play a role in Fe(II) oxidation, as well as sulfide:quinone reductases, are conserved at 82% and 83% AASim. Succinate dehydrogenases, which are well conserved in gene synteny to respective genes in PV-1 only share 65% AASim. This indicates that despite shared geographical origin, similar adaptation strategies, and parallels in metabolic potential, *Zetaproteobacteria* genomes may still differ significantly, for instance in % ANI. Knowledge about how much metabolic potential can vary between *Zetaproteobacteria* of different geographical origin and phylogenetic affiliation, and about their global role in Fe(II) oxidation, will become available upon the expansion of the *Zetaproteobacteria* (meta-)genome database.

## Conflict of Interest Statement

The authors declare that the research was conducted in the absence of any commercial or financial relationships that could be construed as a potential conflict of interest.
